# Further host-genomic characterization of total antibody response to PRRSV vaccination and its relationship with reproductive performance in commercial sows: genome-wide haplotype and zygosity analyses

**DOI:** 10.1186/s12711-021-00676-5

**Published:** 2021-12-07

**Authors:** Leticia P. Sanglard, Yijian Huang, Kent A. Gray, Daniel C. L. Linhares, Jack C. M. Dekkers, Megan C. Niederwerder, Rohan L. Fernando, Nick V. L. Serão

**Affiliations:** 1 Department of Animal Science, Iowa State University, Ames, IA 50011 USA; 2 Smithfield Premium Genetic, Rose Hill, NC 28458 USA; 3 Department of Veterinary Diagnostic & Production Animal Medicine, Iowa State University, Ames, IA 50011 USA; 4 Department of Diagnostic Medicine/Pathobiology, Kansas State University, Manhattan, KS 66506 USA

## Abstract

**Background:**

The possibility of using antibody response (S/P ratio) to PRRSV vaccination measured in crossbred commercial gilts as a genetic indicator for reproductive performance in vaccinated crossbred sows has motivated further studies of the genomic basis of this trait. In this study, we investigated the association of haplotypes and runs of homozygosity (ROH) and heterozygosity (ROHet) with S/P ratio and their impact on reproductive performance.

**Results:**

There was no association (*P*-value ≥ 0.18) of S/P ratio with the percentage of ROH or ROHet, or with the percentage of heterozygosity across the whole genome or in the major histocompatibility complex (MHC) region. However, specific ROH and ROHet regions were significantly associated (*P*-value ≤ 0.01) with S/P ratio on chromosomes 1, 4, 5, 7, 10, 11, 13, and 17 but not (*P*-value ≥ 0.10) with reproductive performance. With the haplotype-based genome-wide association study (GWAS), additional genomic regions associated with S/P ratio were identified on chromosomes 4, 7, and 9. These regions harbor immune-related genes, such as *SLA-DOB*, *TAP2*, *TAPBP*, *TMIGD3*, and *ADORA*. Four haplotypes at the identified region on chromosome 7 were also associated with multiple reproductive traits. A haplotype significantly associated with S/P ratio that is located in the MHC region may be in stronger linkage disequilibrium (LD) with the quantitative trait loci (QTL) than the previously identified single nucleotide polymorphism (SNP) (H3GA0020505) given the larger estimate of genetic variance explained by the haplotype than by the SNP.

**Conclusions:**

Specific ROH and ROHet regions were significantly associated with S/P ratio. The haplotype-based GWAS identified novel QTL for S/P ratio on chromosomes 4, 7, and 9 and confirmed the presence of at least one QTL in the MHC region. The chromosome 7 region was also associated with reproductive performance. These results narrow the search for causal genes in this region and suggest *SLA-DOB* and *TAP2* as potential candidate genes associated with S/P ratio on chromosome 7. These results provide additional opportunities for marker-assisted selection and genomic selection for S/P ratio as genetic indicator for litter size in commercial pig populations.

**Supplementary Information:**

The online version contains supplementary material available at 10.1186/s12711-021-00676-5.

## Background

Porcine Reproductive and Respiratory Syndrome (PRRS) virus (PRRSV) is one of the most important swine pathogens worldwide, causing an estimated loss of approximately USD 664 million to the United States (US) swine industry per year [1]. PRRS is characterized by respiratory clinical signs in growing pigs and reproductive failure in sows [2]. Strategies such as high biosecurity and vaccination have been implemented to reduce the impact of PRRSV. Although the available PRRSV vaccines do not provide complete protection [3], they have been adopted by many producers to decrease the clinical signs and economic losses caused by this disease.

Total antibody response to PRRSV vaccination, measured as sample-to-positive (S/P) ratio, is a promising genetic indicator for reproductive performance in vaccinated crossbred commercial animals. Studies have shown that S/P ratio to PRRSV vaccination in crossbred gilts has a sizable heritability (0.38) [4, 5] and favorable genetic correlations with litter size traits in non- PRRSV-infected crossbred and purebred sows [5, 6]. Thus, the possibility of selecting for this trait has motivated the study of its genomic basis.

The major histocompatibility complex (MHC; known as swine leukocyte antigen region—SLA) region on *Sus scrofa* chromosome (SSC) 7 has been identified as including a major QTL associated with S/P ratio to PRRSV vaccination. Sanglard et al. [5], using a single nucleotide polymorphism (SNP)-based genome-wide association study (GWAS), found a SNP that explained approximately 30% of the genetic variance of this trait. This genomic region has also been identified to be associated with S/P ratio following natural PRRSV infection in other studies [7–9]. Apart from this region, the quantitative trait loci (QTL) that explain the genetic variance of this trait are spread out across the genome and have small effects. Therefore, further investigation of the genomic basis of S/P ratio to PRRSV could identify novel regions capturing part of the genetic variation not previously identified for this trait.

On the one hand, analyses of runs of homozygosity (ROH) have been a helpful strategy to detect regions under selection and variants associated with traits of interest [10, 11]. Genetic variants that are associated with increased risk of diseases are more likely to be recessive than dominant [12]. Thus, association analyses using ROH have been shown to be a powerful strategy to identify genomic regions associated with diseases in humans [13, 14] and livestock [15, 16]. On the other hand, runs of heterozygosity (ROHet), also known as heterozygosity-rich regions, have been used to identify genomic regions that are under balancing or negative selection, such as those subjected to introgression or admixture, or that are hypervariable [17]. Although S/P ratio to PRRSV vaccination is not under direct selection, this trait is genetically correlated with traits that are under selection [5]. Therefore, ROH and ROHet analyses can be useful to identify novel genomic regions associated with S/P ratio to PRRSV vaccination.

Analyses using ROH and ROHet have been primarily focused on purebred populations. However, Howard et al. [18] reported that ROH from parental lines that share haplotypes can persist in F1 crossbred animals, indicating that crossbred animals can be inbred for a portion of the genome. Thus, the presence of ROH in crossbreds may indicate that the two parental breeds have been selected for the same genomic region or share common ancestral haplotypes. Indeed, Zanella et al. [19] showed that maternal Landrace and Large White lines share common haplotypes and, thus, identical chromosomal segments can be passed down to their crossbred progeny. Although both breeds were developed as separate populations hundreds of years ago, there is evidence from human populations that ancestral haplotypes can persist through several generations in regions with low recombination rates [20, 21]. The generation of S/P ratio data requires that animals are PRRSV-vaccinated, which is done in the commercial (i.e., crossbred) animals and not in the nucleus (i.e., purebred) animals due to the health status of elite animals; thus, associations of ROH and ROHet regions with this trait requires the use of crossbred animals.

Haplotype-based analysis can improve QTL detection compared to SNP-based GWAS, since the information from multiple SNPs is combined by taking advantage of linkage disequilibrium (LD) [22, 23]. Haplotype-based analysis can capture the effects of low-frequency variants in regions, which are often in weak LD with the common SNPs that are preferably included on most genotyping arrays [22, 23]. Therefore, haplotype-based GWAS can provide new insights on the genomic variation of S/P ratio to PRRSV vaccination that was not previously captured by SNP-based approaches.

In this study, we performed additional novel genomic analyses for S/P ratio in a crossbred swine population, including associations of ROH, ROHet and haplotypes with S/P ratio to PRRSV vaccination. We hypothesized that animals with longer ROH (i.e., inbreeding) would have a lower immune response (i.e., S/P ratio), and a positive relationship between the extent of ROHet and immune response. Additional genomic variants would be identified in the haplotype-based GWAS in association with S/P ratio. Finally, we hypothesized that the genomic regions (ROH, ROHet, or haplotypes) associated with S/P ratio would also be associated with reproductive performance due to the genetic correlation between these traits. Hence, the identified regions for S/P ratio were tested for association with reproductive performance.

## Methods

All methods described in this study were approved by the Institutional Animal Care and Use Committee at Iowa State University (IACUC# 6-17-8551-S).

### Animals and phenotypic and genotypic data

A complete description of the data used in this study is in Sanglard et al. [5]. Briefly, 906 naïve F1 (Landrace × Large White) replacement gilts at 139 ± 17 days of age from two commercial farms in North Carolina, USA, were vaccinated intramuscularly with a commercial modified live PRRSV vaccine (Ingelvac PRRS® MLV, Boehringer Ingelheim Animal Health, Ames, IA, USA). Blood samples were taken on days 52 and 53 after vaccination for one farm and on day 46 for the other farm. The dates of collection within farms were considered as different contemporary groups (CG), i.e., CG 1, 2, and 3, respectively. Blood samples were used for measurement of immunoglobulin G (total antibody response) against PRRSV, as sample-to-positive (S/P) ratio, using a commercial ELISA test (IDEXX PRRS X3, IDEXX Laboratories Inc., Westbrook, ME, USA). The blood sample of each animal was placed on blood cards for genotyping using the GGP Porcine HD (Neogen GeneSeek, Lincoln, NE, USA) for 50,697 SNPs. The quality control included setting genotypes to missing if the GC score was lower than 0.50, removing SNPs with a call rate lower than 0.90, and removing animals with a genotype call rate lower than 0.90. After filtering, no individuals were removed, and the final dataset used for subsequent analyses included 45,536 SNPs and 906 individuals. Missing genotypes were imputed using IMPUTE2 [24]. The positions of SNPs on the genome were based on the *Sus scrofa* 11.1 assembly.

A subset of 887 of these animals had their reproductive performance recorded for up to three parities [5] from January 2018 (~ 150 days after blood collection) to December 2018 for number born alive (NBA), number of stillborn (NSB), number born mummified (MUM), number born dead (NBD; NSB + MUM), and total number born (TNB; NBA + NBD).

### Homozygosity and heterozygosity

The relationship between homozygosity and heterozygosity with S/P ratio was explored by association analyses with ROH and ROHet regions and with the percentage of the genome consisting of ROH and ROHet (%ROH and %ROHet, respectively). Regions along the genome containing ROH and ROHet were identified with the package *DetectRuns* [25] in the R software [26]. The default values from this package were used for most parameters, with the exception of a minimum of 20 SNPs per window, which was based on previous studies [27, 28]. To define a ROH/ROHet for each individual, a sliding window of 20 SNPs was scanned, allowing one possible heterozygous (for ROH analyses) or homozygous (for ROHet analyses) genotype (to account for potential errors in genotyping and imputation), with a minimum length of 1 Mb and a minimum density of 1 SNP/50 kb.

Then, the ROH or ROHet identified for each animal were aligned across all individuals based on their genomic location, and all the consensus (i.e., overlapping) ROH/ROHet segments were identified. This step resulted in the identification of 3859 and 920 preliminary ROH and ROHet regions, respectively. These regions were numbered according to the number of animals containing a given ROH/ROHet region, with ROH1 and ROHet1 having the largest number of individuals, whereas the last regions, ROH3859 and ROHet920, had the smallest number of individuals. The number and length (in kb) of all preliminary ROH/ROHet obtained for each animal are in Additional file 1: Table S1. These preliminary ROH/ROHet regions were subjected to quality control to remove the short and lowly frequent ones, by filtering out those with less than five SNPs or present in less than 5% of the individuals. After filtering, 511 ROH and 259 ROHet regions were used for association analyses.

The proportion of the genome in ROH (%ROH) and ROHet (%ROHet) was assessed in three ways: %ROH and %ROHet (i) across the whole genome, (ii) on SSC7, and (iii) in the MHC region (SSC7, 22.6–25.2 Mb; based on previous results by Sanglard et al. [5]). In other words, for each individual, these were calculated as the length of ROH/ROHet regions divided by 2.6 Gb, 122 Mb, and 2.5 Mb, for the whole genome, SSC7, and MHC, respectively. The %ROHet was not calculated for the MHC region because no ROHet were identified in this region.

In addition to %ROH and %ROHet, the homozygosity and heterozygosity of individuals were assessed based on SNP genotypes. The proportion of heterozygosity (%Het) of an individual was estimated as the percentage of genotyped SNPs that were heterozygous, noting that the percentage of homozygotes is simply 100%Het. The %Het has the disadvantage that it does not distinguish between identity-by-state and identity-by-descent [29]. However, methods based on ROH and ROHet depend highly on the accuracy of the SNP map. Although the *Sus scrofa* 11.1 assembly has provided substantial coverage of the genome, it is not complete and likely contains errors, especially in the MHC region [30]. Thus, we also investigated this approach to verify the possible relationship of S/P ratio with %Het across the whole genome, on SSC7, and in the MHC region.

### Haplotype construction

SNP genotypes were phased using the Eagle v2.4.1 software [31]. Haplotype blocks were identified by PLINK version 1.90b5.3 [32] based on the default pairwise LD for SNPs within a 200‐kb window. This value was also used because of the rapid decay in pairwise SNP LD expected in crossbred compared to purebred populations [33]. Pairs of SNPs were considered to be in strong LD when the upper boundary confidence interval of D′ was ≥ 0.98 and the lower boundary was ≥ 0.7 [34]. The haplotype blocks were ordered based on the location in the genome (Haplo1 to Haplo5399).

### Statistical analyses

#### Associations of homozygosity and heterozygosity with S/P ratio

Associations of the different measures of homozygosity and heterozygosity with S/P ratio were assessed by fitting the following model:

Model 1 $${\mathbf{y}} = {\upmu } + {\mathbf{Xb}} + {\mathbf{Zu}} + {\mathbf{e}},$$

where ${\mathbf{y}}$ is the vector of phenotypes (S/P ratio), $\upmu$ is the overall mean, ${\mathbf{X}}$ is the incidence matrix relating the fixed effects to the phenotypes, $\mathbf{b}$ is the vector of fixed effects (CG + fixed effects described below), ${\mathbf{Z}}$ is the incidence matrix relating the random effects to the phenotypes, ${\mathbf{u}}$ is the vector of random animal genetic effects, with ${\mathbf{u}}\sim N({\mathbf{0}}, {\mathbf{GRM}}{\sigma }_{u}^{2}),$ where ${\mathbf{GRM}}$ is the genomic relationship matrix (based on Method I of VanRaden [35]), and $\mathbf{e}$ is the vector of random residual effects, with $\mathbf{e}\sim \text{N}({\mathbf{0}},\mathbf{I}{\upsigma }_{\text{e}}^{2})$, where $\mathbf{I}$ is the identity matrix.

Model 1 was used to perform five types of analyses for S/P ratio. First, all ROH (n = 511) regions were simultaneously fitted in this model as categorical fixed effects. This approach was also used in a separate analysis for all ROHet (n = 259) regions. These ROH/ROHet categorical fixed effects were coded as “yes” if the individual contained the ROH/ROHet in a given ROH/ROHet region, or “no” otherwise). For the remaining three types of analyses, associations between the percentage of homozygosity and heterozygosity with S/P ratio were performed by fitting %ROH, %ROHet, or %Het as fixed-effect covariate in Model 1, one at a time. Each of these were analyzed three times, according to the genomic regions used to calculate these percentages. The different genomic regions used included the whole genome, and SSC7 and the MHC region.

After identifying significant associations of specific ROH and ROHet regions with S/P ratio, we investigated if these associations were due to the dominance effects of SNPs within these regions. For that, we estimated the effects captured by the SNPs within the significant ROH/ROHet region (Table 1) on S/P ratio. For each ROH or ROHet, the SNPs were simultaneously fitted as categorical effects in Model 1, along with the remaining ROH or ROHet regions. These additional analyses were performed separately for ROH and ROHet. Significant SNPs were then tested for their additive and dominance effects using orthogonal contrasts. Analyses were performed in ASReml v4.0 [36].

**Table 1:** Regions of runs of homozygosity (ROH) and runs of heterozygosity (ROHet) that were significantly associated^a^ with antibody response to PRRSV vaccination

ROH/ROHet	SSC	Start pos (Mb)	End pos (Mb)	*P*-value	# SNPs^b^	Coefficient (SE)^c^
ROH						
ROH1119	1	55.4	56.5	0.0021	26	− 0.28 (0.09)
ROH681	4	70.1	74.1	0.0037	90	− 0.19 (0.07)
ROH1783	5	93.6	95.2	0.0089	49	0.31 (0.12)
ROH2475	5	94.5	98.9	0.0038	102	− 0.42 (0.14)
ROH2949	7	8.4	11.4	0.0005	85	− 0.30 (0.09)
ROH1596	10	57.5	58.9	0.0037	31	− 0.25 (0.09)
ROH2603	11	63.3	64.4	0.0064	29	0.21 (0.08)
ROH1143	13	193.5	194.6	0.0058	32	− 0.15 (0.05)
ROHet						
ROHet133	5	71.2	71.6	0.006	10	0.12 (0.05)
ROHet240	17	2.1	2.8	0.009	17	0.15 (0.06)

*PRRS* Porcine reproductive and respiratory syndrome, *ROH* runs of homozygosity, *ROHet* runs of heterozygosity, *SSC* *Sus scrofa* chromosome, *pos* position

^a^*P*-value < 0.01

^b^Number of SNPs within the ROH or ROHet region

^c^Effect on sample-to-positive (S/P) ratio by presenting the ROH or ROHet

### Haplotype-based GWAS for S/P ratio

**Fig. 1:**
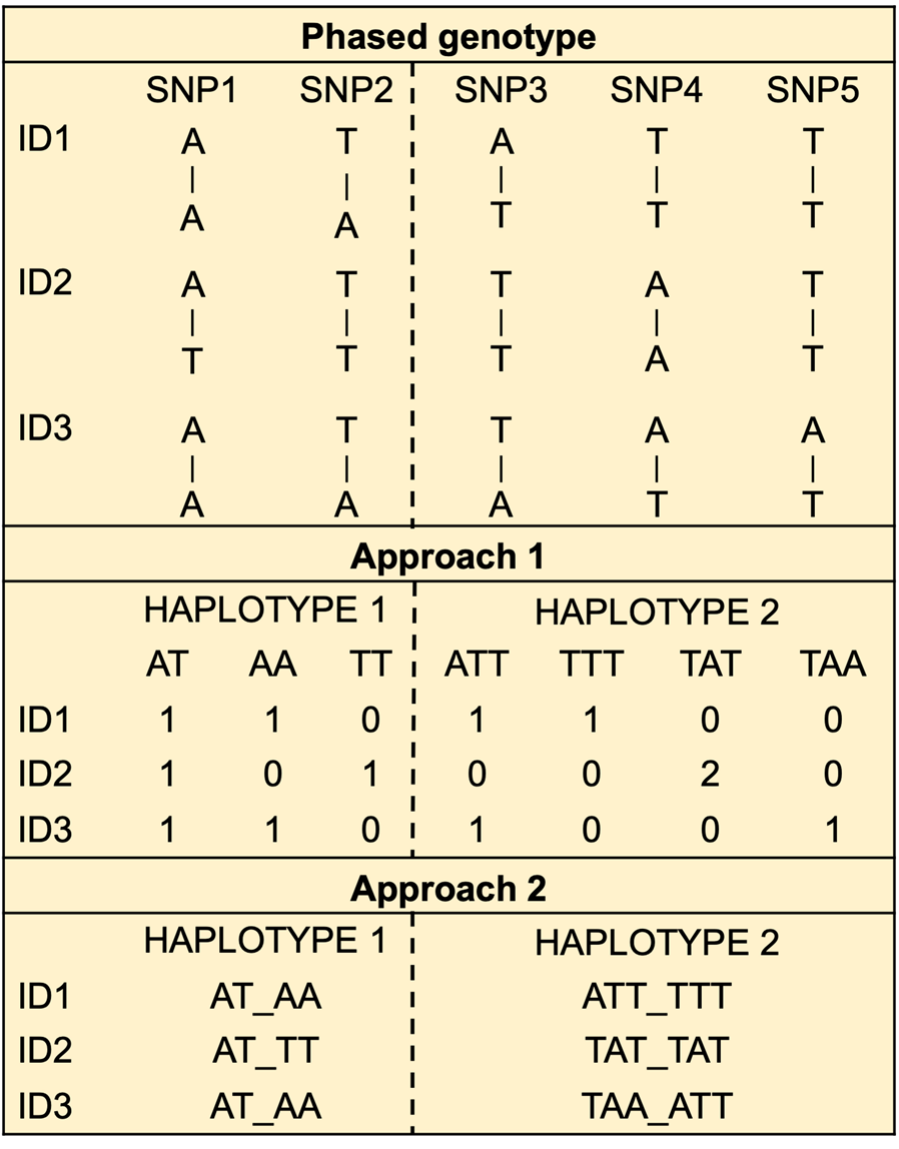
Diagram of how haplotype variants were defined based on phased SNP genotypes using two approaches. In this diagram we have three individuals with five SNPs, for which haplotype 1 is formed by SNPs 1 and 2, and haplotype 2 is formed by SNPs 3, 4, and 5. Approach 1: each haplotype was considered as a variable with the number of copies of each haplotype (i.e., 0, 1, or 2) for each individual as levels. Approach 2: all possible haplotype blocks were considered as a variable with diplotypes as levels. In this approach, rare haplotypes (frequency ≤ 5%) were grouped into a single class

Haplotype-based GWAS was conducted using two approaches. In the first, all haplotypes for each haplotype block across the genome were simultaneously fitted as random effects by including each haplotype across the genome as a different covariate, coded as 0, 1, or 2, based on the number of copies of that haplotype carried by the individual. A small example of how the haplotypes were coded is in Fig. 1 (Approach 1). The haplotype-based GWAS was performed using BayesB (π = 0.99 [37]), with the following model:

Model 2 $${\text{y}}_{{\text{i}}} = {\upmu } + {\text{CG}}_{{\text{k}}} + \mathop \sum \limits_{{{\text{j}} = 1}}^{{\text{m}}} {\text{z}}_{{{\text{ij}}}} {\upalpha }_{{\text{j}}} {\text{d}}_{{\text{j}}} + {\text{e}}_{{\text{i}}} ,$$

where $\upmu$ is the overall mean,${y}_{i}$ is the phenotype value for individual $\text{i}$, ${\text{CG}}_{\text{k}}$ is the fixed effect of the $\text{k}$th contemporary group, $\text{m}$ is the number of haplotypes, ${\text{z}}_{\text{ij}}$ is the number of copies of the $\text{j}$th haplotype (coded as 0, 1, and 2) carried by individual $\text{i}$, ${\upalpha }_{\text{j}}$ is the effect of haplotype $\text{j}$, assuming ${\varvec{\upalpha}}\sim{N}({\mathbf{0}},\mathbf{I}{\upsigma }_{{\upalpha }}^{2})$, ${\text{d}}_{\text{j}}$ is an indicator whether the haplotype $\text{j}$ was included (${\text{d}}_{\text{j}}$ = 1) or not (${\text{d}}_{\text{j}}$ = 0) in the model for a given iteration of the Monte Carlo Markov chain (MCMC), and ${\text{e}}_{\text{i}}$ is the residual associated with the phenotype of individual $\text{i}$*,* with vector $\mathbf{e}\sim{N}({\mathbf{0}},\mathbf{I}{\upsigma }_{\text{e}}^{2})$. Bayesian analyses consisted of 50,000 MCMC, with the first 5000 discarded as burn-in and thinning equal to 100 samples. Haplotypes within 1-Mb windows with a posterior probability of inclusion (PPI) higher than 0.70 [38] are reported. Analyses were performed using the *JWAS* package [39], written in Julia programming language [40].

For the second approach, each haplotype block was fitted separately as a categorical explanatory fixed effect in Model 1, along with CG. In other words, all possible levels (diplotypes) of the haplotype block were used. Because genotypes were phased, the origin of the haplotypes could be differentiated, and the levels of diplotypes were defined by paternal origin followed by maternal origin. Rare diplotypes within a haplotype block (frequency ≤ 5%) were grouped into a single level. A small example of how the haplotype blocks were coded is shown in Fig. 1 (Approach 2). False discovery rate (*q*-value) was used for multiple testing correction [41]. Initially, a *q*-value < 0.05 was used to identify significant association. However, preliminary analyses showed several results at *q*-value < 0.05, only one at 0.05 < *q*-value < 0.1, and the rest at *q*-value > 0.22. Hence, we used a *q*-value < 0.10 as the final threshold for associations.

We performed additional analyses based on preliminary results in this study. Results from both haplotype approaches showed that Haplo2293, located on the MHC region, was significantly associated with S/P ratio. Thus, to better evaluate the complementary effects of Haplo2293 and the H3GA0020505 SNP, identified by Sanglard et al. [5] in a SNP-based GWAS using the same dataset as in this study, we performed three additional GWAS: (1) we fitted the H3GA0020505 SNP as a fixed effect in Model 2 and performed a haplotype-based GWAS; (2) we fitted the three SNPs (H3GA0020505, M1GA0009777, and ASGA0032113) previously identified to be associated with S/P ratio by Sanglard et al. [5] as explanatory random covariates, along with the haplotypes in Approach 1; and (3) we fitted the three haplotypes explaining significantly part of the genetic variance of S/P ratio along with all SNPs used in the univariate GWAS by Sanglard et al. [5] as random covariates in Model 2. All analyses were performed in ASReml v4.0.

#### Association of significant ROH/ROHet and haplotypes with reproductive performance

Previous studies have shown a high genetic correlation of S/P ratio with reproductive performance in PRRSV-vaccinated commercial sows [5]. Thus, to further explore the genomic regions responsible for this correlation, we tested the association of ROH/ROHet regions or haplotype blocks identified for S/P ratio with the reproductive performance of these animals. In other words, the ROH/ROHet regions or haplotypes significantly associated with S/P ratio were tested for reproductive traits using an animal repeatability model. For this, each reproductive trait was analyzed three times as the response variable in a model including the fixed effects of intercept, farm, parity, and one of the following: ROH regions, ROHet regions, or haplotypes blocks (i.e., Approach 2 in Fig. 1, in which haplotypes are fitted in the model with their diplotypes as levels). In addition, the model included the random effects of month/year of farrow ($\text{MYF}$), assumed to be distributed as $\sim {N}({\mathbf{0}}, \mathbf{I}{\upsigma }_{\text{MYF}}^{2})$, permanent environment effects ($\text{pe}$), to account for multiple records of the same sow, assumed to be distributed as $\sim {N}({\mathbf{0}}, \mathbf{I}{\upsigma }_{\text{pe}}^{2})$, and the animal genetic effects ($\text{u}$), assumed to be distributed as $\sim{N}({\mathbf{0}}, \mathbf{G}\mathbf{R}\mathbf{M}{\upsigma }_{\text{u}}^{2})$. Analyses were performed in ASReml v4.0.

## Results

### Homozygosity and heterozygosity

**Fig. 2:**
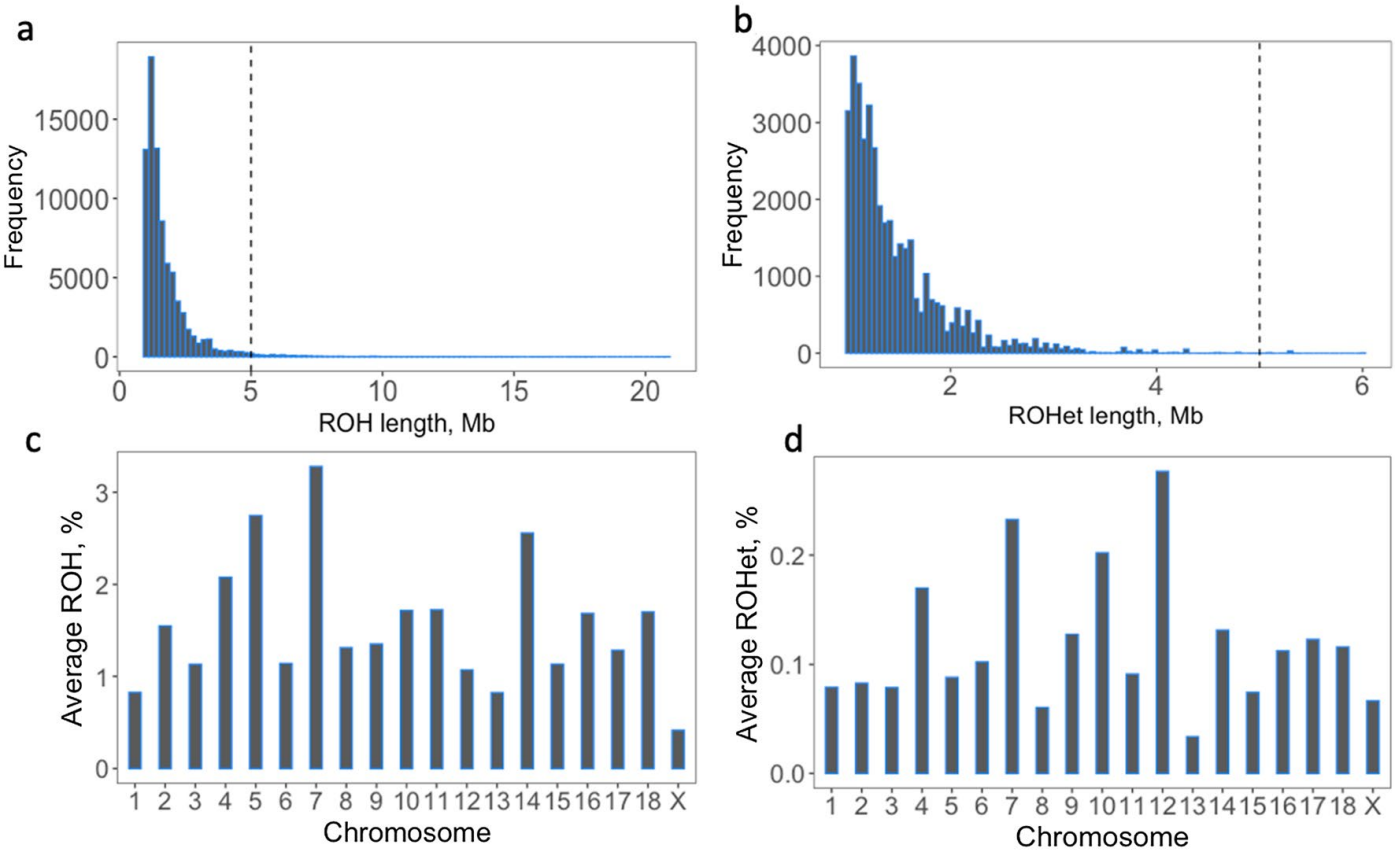
Characterization of runs of homozygosity (ROH; a and c) and runs of heterozygosity (ROHet; **b** and **d**). Dashed lines in **a** and **b** represent 5 Mb. Average proportion lengths of ROH and ROHet by chromosome, in %, in **c** and **d**, respectively. The x-axis in **c** and **d** represent the chromosome

**Fig. 3:**
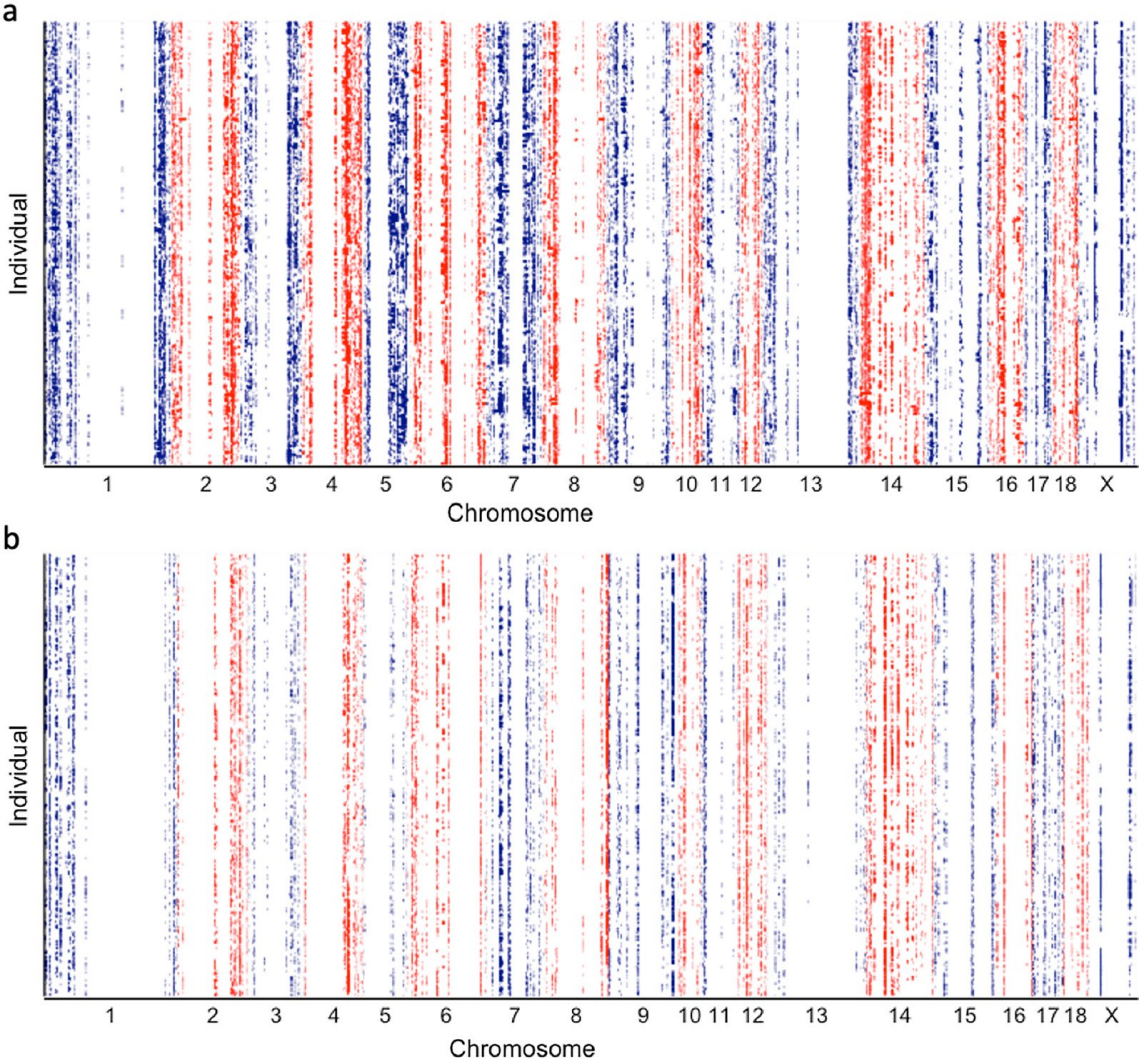
Distribution of runs of homozygosity (ROH; **a**) and runs of heterozygosity (ROHet; **b**) across individuals (y-axis) by chromosome (x-axis). Individuals on the y-axis are clustered based on Ward’s hierarchical clustering analysis. The x-axis is sorted by genome location within chromosome. The colors represent each chromosome, alternating between blue and red

In total, 3859 ROH and 920 ROHet were identified across the genome (see Additional file 1: Table S1). After filtering for ROH and ROHet regions that have at least five SNPs and 45 individuals (i.e., 5% of the samples), 511 ROH and 259 ROHet were used in the association analyses. On average, there were 52 ± 32 SNPs per ROH and 36 ± 13 SNPs per ROHet. Most ROH (Fig. 2a) and ROHet (Fig. 2b) were short (≤ 5 Mb). SSC7 and 12 on average had the highest %ROH (Fig. 2c; 3.27%) and %ROHet (Fig. 2d; 0.27%) across individuals, respectively, and SSCX and 13 had the lowest %ROH (Fig. 2c; 0.41%) and %ROHet (Fig. 2d; 0.03%), respectively. The lower %ROH observed on the X chromosome may be due to the lower density of SNPs/Mb than in the rest of the genome. For example, the density (i.e., number of SNPs/Mb) on the X chromosome was 24% lower than the average density of the rest of the genome. Distributions of ROH and ROHet across individuals and across each chromosome are shown in Fig. 3a, b, respectively. In general, ROH and ROHet were concentrated at the beginning and end of the chromosomes.

Results for the association analyses of ROH and ROHet regions with S/P ratio are in Table 1 and Additional file 2: Table S2. Eight ROH and two ROHet regions were significantly associated (*P*-value ≤ 0.01) with S/P ratio (Table 1). None of the ROH or ROHet regions that were significant for S/P ratio were associated (*P*-value ≥ 0.10) with reproductive performance in this dataset (data not shown). There was no association (*P*-value ≥ 0.18) of %ROH, %ROHet, or %Het across the whole genome, on SSC7, or in the MHC region with S/P ratio (see Additional file 3: Figure S1).

The results of the additional analyses to identify if the associations of ROH and ROHet regions with S/P ratio were due to dominance effects within these regions showed that eight SNPs within five ROH regions were significantly associated (*P*-value ≤ 0.05) with S/P ratio and showed significant (*P*-value ≤ 0.05) dominance effects (Table 2). In contrast, no SNP within the two ROHet had significant dominance effects (*P*-value > 0.05).

**Table 2:** SNPs within significant ROH^a^ with dominance^b^ effects

ROH	SNP	*P*-value^c^			Estimated effects^d^	
		SNP	Add	Dom	Add	Dom
ROH1783	ALGA0033636	0.03	0.20	0.01	0.15	− 0.22 (0.08)
ROH2475	WU_10.2_5_103352361	0.005	0.02	0.04	1.26	− 0.40 (0.19)
ROH2949	ALGA0038565	0.03	0.04	0.01	− 0.23	0.16 (0.06)
ROH2949	WU_10.2_7_11285030	0.0002	0.01	0.01	− 2.30	− 1.75 (0.68)
ROH1596	WU_10.2_10_64433795	0.01	0.02	0.01	0.70	0.25 (0.23)
ROH1596	WU_10.2_10_64461127	0.04	0.03	0.02	− 0.90	1.07 (0.44)
ROH1596	WU_10.2_10_64672192	0.03	0.0	0.005	− 0.24	− 1.17 (0.44)
ROH2306	ASGA0051263	0.04	0.16	0.04	0.72	− 0.98 (0.49)

*Add* additive effect, *Dom* dominance effect

^a^ROH associated with sample-to-positive (S/P) ratio are presented in Table 1

^b^Only significant (*P*-value < 0.05) SNPs showing dominance effects (*P*-value < 0.05) are included in this table

^c^*P*-values for the main effect (SNP) of SNP fitted as a categorical effect. SNPs with significant (*P*-value < 0.05) main effect were subjected to orthogonal contrasts analysis to test their respective additive (Add) and dominance (Dom) effects

^d^Estimates for the additive (Add) and dominance (Dom) effects of each SNP. Add effects represent the effect of number of each B allele. Standard errors are between parentheses

### Haplotype analyses

In total, 5399 haplotype blocks were identified across the genome, with on average 3.7 ± 1.7 SNPs per haplotype block, ranging from 2 to 13 SNPs. The average haplotype block length was 86.2 ± 73.8 kb, ranging from 0.0002 to 199.9 kb. At least one haplotype block was identified for each chromosome. A summary of all haplotype blocks is in Additional file 4: Table S3.

**Fig. 4:**
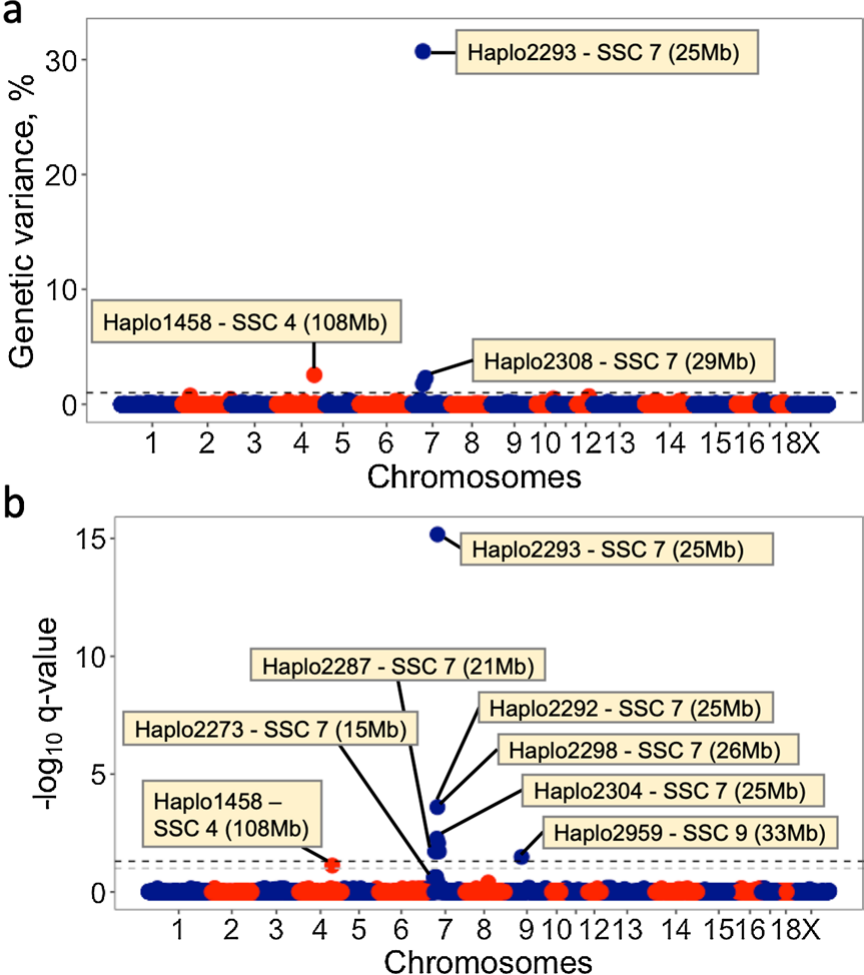
Haplotype GWAS based on approaches 1 (**a**) and 2 (**b**) for sample-to-positive (S/P) ratio. In **a**, the y-axis corresponds to the total genetic variance explained by the haplotypes in percentage, whereas in **b**, the y-axis corresponds to the *q*-value in -log_10_ scale. For both plots, the x-axis corresponds to the chromosome location of the haplotypes. Significant associations [PPI > 0.70 in **a** and *q*-value < 0.10 in **b**] are labeled on the plot. In **b**, the black and grey dashed lines correspond to *q*-value thresholds of 0.05 and 0.10, respectively

**Fig. 5:**
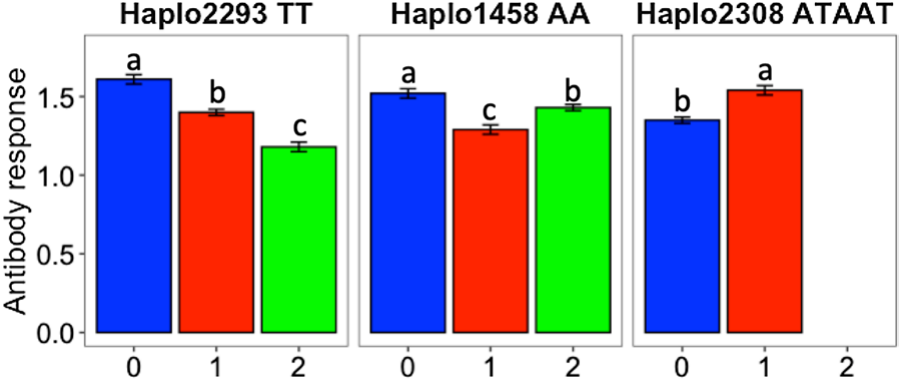
Expected means of antibody response [sample-to-positive (S/P) ratio] to porcine reproductive and respiratory syndrome virus vaccination for each significant haplotype (*P*-value < 0.001) based on Approach 1

Based on the first approach of fitting all possible haplotypes across the genome simultaneously as random effects, three haplotypes were associated (posterior probability of inclusion; PPI ≥ 0.70) with S/P ratio (Fig. 4a and Table 3). These three haplotypes were then fitted simultaneously in Model 1 as fixed effects, with results presented in Fig. 5. All three haplotypes were still significantly (*P*-value < 0.001) associated with S/P ratio. For Haplo2293 (haplotype TT), there was a clear additive effect, with a reduction in S/P ratio as the number of copies of TT increased (*P*-value ≤ 0.05). For Haplo1458 (haplotype AA), animals with one copy of AA had a lower S/P ratio (*P*-value ≤ 0.05) than those with zero or two copies of this haplotype. These results indicate partial underdominance of Haplo1458. Finally, for Haplo2308 (haplotype ATAAT), none of the animals had two copies of this haplotype but animals with one copy had a greater (*P*-value ≤ 0.05) S/P ratio than those with no copies.

**Table 3:** Haplotypes associated^a^ with antibody response^b^ to PRRSV based on haplotype GWAS

Approach 1					
Haplotype	SSC	Initial pos (Mb)	End position (Mb)	% genetic variance^c^	PPI
Haplo1458 AA	4	108.884	108.898	2.55	0.82
Haplo2293 TT	7	25.004	25.015	30.72	1.00
Haplo2308 ATAAT	7	29.107	29.268	2.28	0.70

Approach 2					
Haplotype	SSC	Initial pos (Mb)	End position (Mb)	Number of SNPs^d^	*q*-value
Haplo1458	4	108.884	108.898	2	0.076
Haplo2273	7	15.347	15.373	3	0.019
Haplo2287	7	21.166	21.181	3	0.005
Haplo2292	7	24.979	24.986	3	< 0.001
Haplo2293	7	25.004	25.015	2	< 0.001
Haplo2298	7	26.522	26.715	4	0.008
Haplo2304	7	27.708	27.906	5	0.019
Haplo2959	9	33.449	33.561	4	0.033

SSC, *Sus scrofa* chromosome

^a^Posterior probability of inclusion (PPI) > 0.70 (Approach 1) or *q*-value < 0.1 (Approach 2)

^b^Measured as sample-to-positive (S/P) ratio

^c^% genetic variance: percentage of the genetic variance explained by a given haplotype

^d^Number of SNPs in each haplotype

**Fig. 6:**
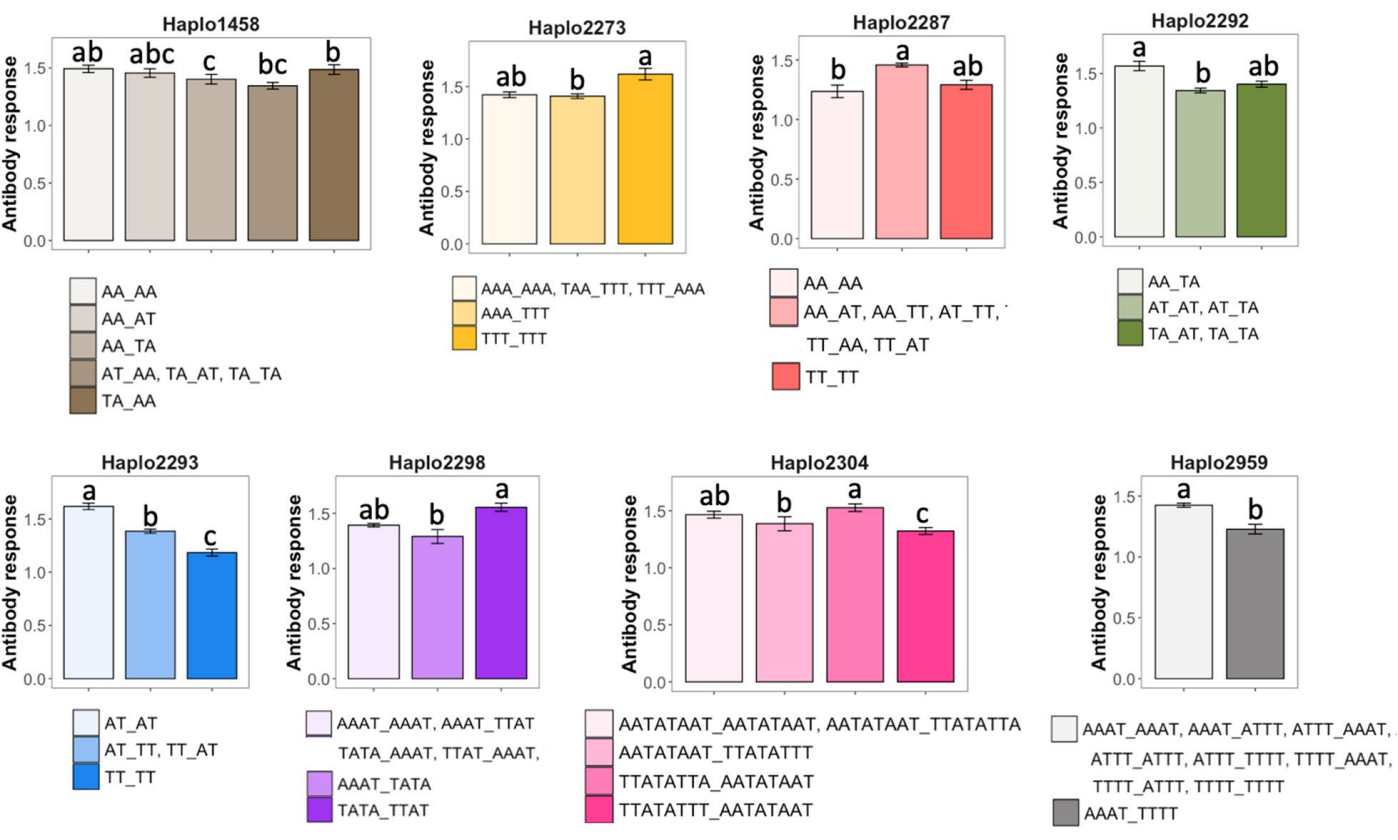
Expected means of antibody response [sample-to-positive (S/P) ratio] to porcine reproductive and respiratory syndrome virus vaccination for each haplotype block significantly (*q*-value ≤ 0.05) associated with S/P ratio based on Approach 2. The different bar transparencies represent diplotypes. Means without the same superscript (a–c) within a haplotype block indicate statistical differences at *P*-value < 0.05. Diplotypes that were not significantly different (*P*-value > 0.05) from others were combined into the same column to facilitate visualization. The order of the haplotypes follows the order of their location along the genome

In the second approach, each haplotype block was fitted separately as a fixed effect in Model 1. Complete results are in Additional file 5: Table S4. Eight haplotype blocks were associated (*q*-value ≤ 0.076) with S/P ratio (Fig. 4b and Table 3). Of these, six haplotype blocks were located on SSC7, including Haplo2293 (*q*-value < 0.001), which was also identified in the first approach (Fig. 2a). Haplo1458 (*q*-value = 0.076), located on SSC4, was also identified using the first approach (Fig. 4a). The other significant haplotype block (Haplo2959; *q*-value = 0.033) was located on SSC9 (~ 33.5 Mb) and included four SNPs (ASGA0094196, MARC0045992, ALGA0108168, and UMB10000140). The expected S/P ratio for each diplotype is shown in Fig. 6. Haplo2293 on SSC7 (25 Mb) had a clear additive effect while all other haplotype blocks had more complex relationships among diplotypes.

Additional GWAS were performed to better understand the relationship between Haplo2293 and the H3GA0020505 SNP, both located in the MHC region. The results revealed that the genetic variance of S/P ratio explained by Haplo2293 dropped from 30.7% (PPI = 1.00) to 11.5% (PPI = 0.81) when the H3GA0020505 SNP (PPI ≥ 0.98) was fitted as a fixed effect. This showed that after accounting for the effect of the H3GA0020505 SNP, Haplo2293 still captured a substantial proportion of the genetic variance, confirming that both loci were able to explain variation in S/P ratio.

**Fig. 7:**
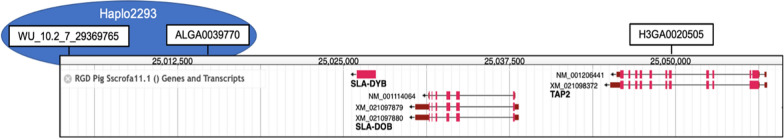
Location of SNPs and candidate genes on chromosome 7. Candidate genes on chromosome 7 (25,004,228 to 25,058,030) between the Haplo 2293 haplotype and the H3GA0020505 SNP that are significantly associated with antibody response to porcine reproductive and respiratory syndrome

By fitting the three SNPs (H3GA0020505, M1GA0009777, and ASGA0032113) along with the haplotypes fitted in Approach 1, we observed that Haplo2293 explained 13.7% of the genetic variance of S/P ratio (PPI = 0.72), Haplo1458 explained 2.8% (PPI = 0.71), Haplo2308 explained 0.1% (PPI = 0.10), the H3GA0020505 SNP explained 6.9% (PPI = 0.68), the M1GA0009777 SNP explained 6.0% (PPI = 0.73), and the ASGA0032113 SNP explained 3.4% (PPI = 0.60). Thus, the genetic variances explained by the H3GA0020505 and ASGA0032113 SNPs were not significant when the haplotypes were fitted in the model. This corroborates the hypothesis that these SNPs and Haplo2293 may be capturing the same QTL. The LD of the H3GA0020505 SNP with the WU_10.2_7_29369765 SNP and with the ALGA0039770 SNP that make up Haplo2293 were *r*^*2*^ = 0.46 and 0.003, respectively. The LD between the ASGA0032113 SNP with the WU_10.2_7_29369765 and ALGA0039770 SNPs of Haplo2293 were *r*^*2*^ = 0.57 and 0.009, respectively. Thus, there is strong evidence that Haplo2293 and the H3GA0020505 SNP capture the effects of a QTL that is located between them (note that the ASGA0032113 SNP is also located between them) (Fig. 7). Another hypothesis is that each haplotype and SNP capture the effects of different QTL that are located between them, and those QTL are in high LD with each other. Either way, Haplo2293 may be in stronger LD with the QTL than the H3GA0020505 SNP, given that Haplo2293 explained more of the genetic variance of S/P ratio. The M1GA0009777 SNP, which is located upstream of these SNPs, most likely captures the effects of a different QTL.

When the three haplotypes explaining a significant part of the genetic variance of S/P ratio were fitted along with all SNPs, Haplo2293 explained 23.5% of the genetic variance of S/P ratio (PPI = 0.98), Haplo1458 explained 1.0% (PPI = 0.56), Haplo2308 explained 0.1% (PPI = 0.11), the H3GA0020505 SNP explained 3.4% (PPI = 0.70), the M1GA0009777 SNP explained 1.2% (PPI = 0.56), and the ASGA0032113 SNP explained 0.1% (PPI = 0.04). Similar to what was observed before, the ASGA0032113 SNP was not significant when fitted along with Haplo2293. Surprisingly, the H3GA0020505 SNP also explained a significant part of the variance, while the M1GA0009777 SNP did not. The estimate of the total genetic variance of S/P ratio when fitting only haplotypes was slightly lower (0.074) than the estimate obtained when all SNPs (0.079) were fitted. This shows that more genetic variance is being captured when fitting all the SNPs than when fitting all the haplotypes. Thus, it may help to justify why when fitting the main haplotypes along with the significant SNPs, both Haplo2293 and the H3GA0020505 SNP were significant. To investigate why the M1GA0009777 SNP was not significant, we estimated the LD of this SNP with the WU_10.2_7_29369765 and ALGA0039770 SNPs that make up Haplo2293 and found it to be essentially zero. Given the very low LD and the substantial distance (~ 1 Mb) between the M1GA0009777 SNP and Haplo2293, it is more likely that they capture a different QTL. However, it is possible that part of the effect of this QTL is also captured by Haplo2293. Thus, the presence of Haplo2293 along with other SNPs capturing a small portion of the genetic variance of the QTL located in this region indicates that the effect of the M1GA0009777 SNP was not significant.

#### S/P ratio haplotypes associated with reproductive performance

The nine haplotype blocks that were associated with S/P ratio were also investigated for associations with reproductive performance traits (Table 4) and see Additional file 6: Table S5. Four haplotype blocks were associated with at least one reproductive performance trait (*P*-value ≤ 0.09). Two haplotype blocks were associated with NBA (*P*-value ≤ 0.09) and TNB (*P*-value ≤ 0.05), and one was associated with NBD (*P*-value = 0.02), while no associations were found for NSB and MUM (*P*-value ≥ 0.12). Thus, some of the haplotype blocks associated with S/P ratio also appear to have an impact on the reproductive performance of crossbred sows.

**Table 4:** *P*-values for the association of S/P ratio haplotypes^a^ with reproductive performance

Haplotype	NBA		TNB	NSB	MUM	NBD
Haplo1458	0.36		0.25	0.78	0.42	0.87
Haplo2273	0.80		0.87	0.13	0.12	*0.02*
Haplo2287	0.42		0.37	0.85	0.21	0.91
Haplo2292	*0.09*		0.38	0.85	0.19	0.40
Haplo2293	0.21		0.44	0.57	0.26	0.37
Haplo2298	0.18		0.18	0.94	0.49	0.79
Haplo2304	*0.04*		*0.04*	0.68	0.43	0.74
Haplo2308	0.15		*0.05*	0.50	0.41	0.80
Haplo2959	0.44		0.50	0.27	0.47	0.39

Significant associations are being considered at *P*-value < 0.10 (highlighted in italics)

NBA/number born alive; TNB/total number born; NSB/number of stillborn; MUM/number of piglets mummified; NBD/ number of piglets born dead

^a^Haplotypes associated with sample-to-positive (S/P) ratio are in Table 2

## Discussion

In this study, we performed novel genomic analyses for S/P ratio in crossbred sows to further characterize the genomic basis of S/P ratio, with the hope of providing additional variants for marker-assisted selection and genomic selection for S/P ratio as a genetic indicator for litter size in commercial pig populations. We hypothesized that increased length of ROH (i.e., inbreeding) would be associated with lower S/P ratio, and ROH located in regions associated with traits under selection and genetically correlated to S/P ratio would be associated with S/P ratio. This hypothesis was partially supported by the association of ROH with S/P ratio. However, although the ROH were located in regions previously associated with traits under selection, ROH regions were not associated with reproductive traits in our study. In addition, in our study we did not identify an association between inbreeding level and S/P ratio. We had also hypothesized that more variants would be identified in association with S/P ratio, and this hypothesis was supported by the identification of the haplotypes on SSC4, 7 (outside the MHC region), and 9.

### Homozygosity and heterozygosity

Most of the ROH and ROHet identified across the genome were short (< 5 Mb), which reflects the pattern expected for a crossbred population. It has been suggested that natural and artificial selection strongly shape genomic ROH patterns in livestock [42], but crossbred populations are not directly under selection. The maternal and paternal lines of the crossbred individuals are expected to have some loci that are fixed for opposite alleles, which increases the heterozygosity in the genome of the offspring, contributing to the disruption of long ROH that may be present in the genome of the parental lines [10]. Furthermore, a positive correlation of 0.70 between the average pairwise LD between SNPs and the length of ROH per chromosome has been reported in domestic and wild pig populations [43]. In our data, the correlation between the average LD (*r*^*2*^_LD_) and the percentage of ROH per chromosome was positive and moderate, at 0.65. Crossbred populations are expected to have a rapid decay of LD [33], which was also observed in our population (data not shown). This helps to explain the small number of ROH found in our study.

Most of the ROH identified in this study were located at the ends of the chromosomes. The distribution of short ROH at the ends of the chromosomes partially agrees with Bosse et al. [44], who observed that shorter ROH were located towards the telomeric regions, while longer ROH are centered in chromosomal regions of low recombination in pigs. However, in our study, few long ROH were identified, and we observed no patterns for which ROH were located across the genome.

Each chromosome had at least one ROH and at least one ROHet region. SSC7 showed the highest average %ROH across individuals. Surprisingly, the MHC region had ROH but no ROHet. The MHC region is expected to have a high level of genetic diversity, which is associated with a greater capacity of the organism to defend itself against a wide variety of pathogens [30]. In humans, ROH in the MHC region have been associated with disease susceptibilities, such as rheumatoid arthritis [14] and schizophrenia [45]. In fact, in our population we observed similar levels of heterozygosity in the MHC regions compared with the rest of the genome (results not shown). The quality of the map of the region could also play a role in the absence of ROHet within the MHC region identified in our population. The current genome map of the MHC region in pigs is based on the *Sscrofa11.1* build, which was obtained by sequencing a Duroc female pig that was not homozygous for the MHC region [30]. This could have resulted in an incomplete and inaccurate assembly of the SLA region, especially since the animals in this study had a genetic makeup of Landrace and Large White pigs. Thus, this could have caused the lack of ROHet detected within the MHC region. Nonetheless, ROHet regions were identified in other parts of the genome. In addition, we visually compared the ROHet and non-ROHet regions and could not find specific reasons for the lack of ROHet within the MHC region in our population.

The proportion of ROH along the genome is an indicator of inbreeding and can also provide information about regions of the genome that have undergone directional selection. In crossbred animals, which are not under direct selection, ROH regions along the genome could be a result of selection for similar traits in the parental lines [18]. In our study, the parental lines were Landrace and Large White pigs, which are mostly selected for similar maternal traits [46]. ROH regions along the genome of crossbred animals can also represent ancestral haplotypes that persist in both parental lines [18].

In our study, the %ROH across the whole genome and in the MHC region were not associated with S/P ratio, which may be because S/P ratio has not been under direct or indirect selection in the parental breeds of this crossbred population. In addition, the stretches of homozygosity resulting from selection in the parental lines were not associated with S/P ratio in the crossbred animals. %ROHet and %Het across the whole genome or in the MHC region were also not associated with S/P ratio. This indicates that, similar to inbreeding, the overall heterozygosity in the genome may not be associated with S/P ratio in crossbred gilts.

Although the %ROH and %ROHet were not associated with S/P ratio, specific ROH and ROHet regions were significantly associated with this trait. The presence of specific ROH regions on SSC1, 4, 5, 7, 10, or 13 was associated with a lower S/P ratio, while a specific ROH region on SSC11 was associated with a higher S/P ratio. Although these regions were not associated with reproductive traits in this study, it is possible that selection for other traits of interest in both of the purebred parental lines resulted in ROH regions in the two parental lines that persisted in the crossbred population. Interestingly, some of these regions have been associated with economically important traits, especially in maternal lines, such as teat traits (SSC11 ~ 60 Mb) [47], number of piglets weaned (SSC1 ~ 58 Mb) [48], number of piglets born alive (SCC5 ~ 87 Mb; SSC10 ~ 58 Mb) [49], and total number of piglets born (SSC10 ~ 57 Mb; SSC11 64 Mb) [49]. The presence of ROHet on SSC5 or 17 was associated with a higher S/P ratio. This is in agreement with previous studies that have shown that heterozygosity at genes that are involved in pathogen resistance increases fitness [50, 51]. More specifically, the *ubiquitin specific peptidase 18* (*USP18*) gene, which is associated with innate immunity to viral infection is a potential candidate gene located in this region on SSC5 [52, 53]. Few other genes were identified around these ROHet regions, with non-specific or unrelated functions.

For the dominance effects within ROH and ROHet regions, we hypothesize that association of the presence of ROH regions with a lower S/P ratio is due to the positive (over)dominance effects of some SNPs within these regions. Likewise, the association of the presence of a ROH region with a higher S/P ratio could be due to the negative (under)dominance effects of SNPs in these regions. In fact, the negative effects associated with the presence of ROH2949 and ROH1596 on S/P ratio could be partially explained by the significant positive dominance effects of SNP ALGA0038565 for ROH2949, and of SNPs WU_10.2_10_64433795 and WU_10.2_10_64461127 for ROH1596. Likewise, the positive effects associated with the presence of ROH1783 and ROH2306 on S/P ratio could be partially explained by the significant negative dominance effects of SNP ALGA0033636 for ROH1783 and of SNP ASGA0051263 for ROH2306. In contrast, few SNPs (WU_10.2_5_103352361, WU_10.2_7_11285030, and WU_10.2_10_64672192) had negative dominance effects within ROH that had negative associations with S/P ratio (ROH2475, ROH2949, and ROH1596, respectively). Thus, this contradictory result could be driven by the additive effects and genotype frequencies of other SNPs within these ROH. In summary, the additive and dominance effects and genotype frequencies of SNPs within ROH partially explained the negative or positive associations of the respective ROH with S/P ratio. However, it is still important to note that these SNPs do not represent all the SNPs within a ROH region, and other QTL that are not captured by these SNPs may also impact the effect of a given ROH region. Finally, these results further support that ROH analyses investigate the association with stretches of homozygosity and not necessarily the dominance effect of QTL being captured by individual SNPs.

### Identification of haplotypes

The number of haplotype blocks identified in our study (5399) was larger than the number of haplotype blocks previously reported in four different European crossbred lines, for which the number of haplotype blocks ranged from 3037 to 2649 [54]. It is important to note that these studies are not directly comparable. Although the same algorithm to obtain the haplotype blocks was used in our study and in Veroneze et al. [54], the latter used a threshold of at least three SNPs (we used 2). In fact, the number of haplotype blocks with at least three SNPs was 3194. However, the number of haplotype blocks identified in our population was smaller than the number of haplotype blocks (21,296) identified in a Large White population [55]. LD is expected to be lower in crossbred populations and, consequently, the number of haplotype blocks is also expected to be smaller than in purebred lines [33]. The success of GWAS depends, among other factors, on the level of LD between markers and the causal polymorphisms [56]. Hence, a smaller number of SNP associations is expected to be observed in crossbred than in purebred populations. However, due to the shorter stretches of LD, the variants identified in crossbred animals are expected to be in closer proximity to the causal polymorphisms than in purebred populations [33].

### Haplotype-based GWAS

The main haplotype associated (PPI ≥ 0.70; *q*-value < 0.05) with S/P ratio was Haplo2293. Haplo2293 is located in the MHC class II region (SSC 7 25,004,228–25,014,857 kb). Interestingly, the SNPs that form this haplotype block, WU_10.2_7_29369765 and ALGA0039770, were not significantly associated with S/P ratio in the SNP-based GWAS [5] and explained less than 0.01% of the genetic variance (data not shown) of S/P ratio. This indicates that the combination of the alleles of those SNPs is associated with the QTL. This haplotype block is positioned close to the H3GA0020505 SNP (25,049,757 kb), which has been associated with S/P ratio using the same data from this study [5]. However, the LD between the haplotype block and this SNP was moderate to weak (*r*^*2*^ ≤ 0.45). Given the low LD in the MHC region in this population (*r*^*2*^ = 0.08), these markers might be close to the QTL. This region (Fig. 7) includes the pseudogenes *MHC class II DY/DQ beta-like pseudogene* (*SLA-DYB*) and *MHC class II DO beta* (*SLA-DOB*), and the gene *transporter 2 ATP binding cassette subfamily B member* (*TAP2*). The *SLA-DOB* is involved in the epitope loading of MHC Class II molecules and is associated with both CD4^+^ and CD8^+^ T-cells. In addition, *SLA-DOB* shows a highly significant association with immunoglobulin G and PRRSV-specific antibody response [57]. TAP2 is a transporter that is associated with antigen processing [58]. The TAP2 protein connects with the TAP1 protein to form a complex located in the endoplasmic reticulum membrane, which transports protein fragments (i.e., peptides) from pathogens into the endoplasmic reticulum. These peptides are then attached to the MHC class I proteins and move to the surface of the cell to be presented to specialized immune system cells [59]. MHC class I is responsible for presenting endogenous pathogens, such as viruses, to cytotoxic T-cells (CD8^+^) and has been shown to be downregulated after PRRSV infection [60, 61]. However, PRRSV impairs dendritic cells, which are antigen-presenting, by downregulating the MHC class II expression and limiting the proliferation of leukocytes [62–64]. Therefore, *SLA-DOB* and *TAP2* are strong candidate genes within this region that is associated with S/P ratio. Other genes around this region have also been associated with PRRSV infection, as reviewed in Sanglard et al. [5]. However, the stretch of low LD in this region suggests that Haplo2293 and the H3GA0020505 SNP are close to the causal mutation(s) within the MHC class II region that affects S/P ratio, which supports the possibility that the QTL is located between these two markers. For Haplo2293, the diplotype that lacks haplotype TT (AT_AT) had a greater S/P ratio, followed by diplotypes with only one copy of haplotype TT (AT_TT or TT_AT), while diplotype TT_TT had the lowest S/P ratio. This supports that haplotype TT is associated with a lower S/P ratio.

From the remaining haplotype blocks, six were located on SSC7 and one on SSC9. Among those located on SSC7, Haplo2292, Haplo2298, Haplo2304, and Haplo2308 are located upstream (Haplo2292) and downstream (Haplo2298, Haplo2304, and Haplo2308) of Haplo2293 and most likely capture the same QTL around this region. In fact, after fitting all significant haplotype blocks simultaneously in the model, Haplo2292 (*P*-value = 0.75), Haplo2298 (*P*-value = 0.57), and Haplo2304 (*P*-value = 0.65) were not significant, while Haplo2293 had a highly significant (*P*-value ≤ 0.001) association with S/P ratio. Thus, Haplo2293 may be in stronger LD with the QTL in this region than the other two haplotype blocks. Unlike Approach 1, in Approach 2 each haplotype block was fitted in the model individually and, thus, more than one haplotype block could have captured the same QTL, especially those on SSC7. Haplo2308 is also located on SSC7 (~ 29.2 Mb) and explained a considerable amount of the genetic variance in S/P ratio (~ 2%). The *collagen type XXI alpha 1 chain* gene (*COL21A1*) is the only gene located within this haplotype. Interestingly, the *TAP binding protein* gene (*TAPBP*) is located slightly downstream of this haplotype (~ 29.7 Mb) and is a potential candidate gene for the genetic variance that was captured by this haplotype. Also, on SSC7, Haplo2287 (21 Mb) was significantly associated with S/P ratio even when fitting all significant haplotypes simultaneously. This suggests that another QTL may be located upstream of the MHC region.

For Haplo2287, the homozygous diplotypes (AA_AA and TT_TT) had a lower S/P ratio than the heterozygous diplotypes (AA_AT, AA_TT, AT_TT, TT_AA, and TT_AT). Interestingly, in another region on SSC7 (~ 15.3 Mb), Haplo2273 was also associated with S/P ratio. However, after fitting all SNPs and all significant haplotype blocks simultaneously, this haplotype was, not significant anymore (*P*-value = 0.27). Thus, it is possible that, although they are located far apart (~ 5 Mb), Haplo2273 may capture the same QTL as Haplo2287. Few genes are located in this region on SSC7 (~ 15.3 Mb), and most of them are uncharacterized. The region around Haplo2287 includes several genes that are associated with chromatin modifications: the *H2B clustered histone 1*gene (*H2BC1*), the *H2A clustered histone 6* gene (*H2AC6*), the *H1.4 linker histone*, *cluster member* gene (*H1-4*), the *histone cluster 1 H2bd* gene (*HIST1H2BD*), and the *histone H2B type 1-M* (*H2BC14*) gene.

The region around Haplo1458 on SSC4 (~ 108.8 Mb) contains several relevant genes, including the *transmembrane and immunoglobulin domain containing 3* gene (*TMIGD3*), the *adenosine A3 receptor* gene (*ADORA3*), the *WD repeat domain 77* gene (*WDR77*), the *pepsinogen B* gene (*PGB*), and the *ATP synthase peripheral stalk-membrane subunit beta* gene (*ATP5PB*). Among these, *TMIGD3* and *ADORA3* are potential immune-related candidate genes associated with S/P ratio. SSC4 has been reported to harbor a major QTL for PRRSV resistance (known as *GBP5*) in nursery pigs artificially infected with PRRSV [65] but this region was located downstream (~ 127–128 Mb) of the QTL identified here. For Haplo1458, individuals with two AA haplotypes had a higher S/P ratio than those with only one or no AA haplotypes. Finally, Haplo2959 was located on SSC9 (~ 33.4 Mb), where several matrix metallopeptidase (MMP) genes are located, such as *MMP3*, *MMP8*, *MMP12*, and *MMP27*, which are involved in the process of vascular invasion and apoptosis [66].

In summary, Haplo2293 and the H3GA0020505 SNP seem to be capturing one or several QTL located between them. In addition, the LD of the QTL with Haplo2293 may be stronger than that with the H3GA0020505 SNP. Apart from the QTL located in the MHC class II region, two other major QTL were identified as associated with S/P ratio, which suggests that genes associated with peptide transport are potential candidate genes associated with this trait.

#### S/P ratio haplotypes associated with reproductive performance

Number born alive and TNB had a larger number of associations with haplotype blocks that had been previously associated with S/P ratio than the other reproductive traits. The haplotype blocks that were also significantly associated with reproductive performance were located on SSC7 (Haplo2292, Haplo2304, and Haplo2308). Haplo2292 (~ 25 Mb) is located upstream, and Haplo2298 and Haplo2304 (~ 27.7–29 Mb), downstream of Haplo2293.

The MHC region has also been identified to be important in a bivariate GWAS for S/P ratio and NBA using the same data as in this study, and several potential candidate genes were suggested [5]. We also tried this strategy by performing bivariate haplotype-based GWAS with these data. However, as previously observed in Sanglard et al. [5], few associations were identified (results not shown), probably due to the reduced statistical power of using a relatively small number of individuals and the low heritably of reproductive traits. Nonetheless, the results were similar to our findings, i.e., that the genetic variances and covariance of S/P ratio and reproductive traits were concentrated in the MHC region. Therefore, our haplotype bivariate GWAS has not provided new results.

Interestingly, the diplotype AA_AT (Haplo2292) was also associated with a larger NBA and higher S/P ratio than the diplotypes AT_AT and TA_TA. For Haplo2304, the diplotype TTATATTA_AATATAAT was associated with larger NBA, TNB, and higher S/P ratio than the diplotype TTATATTT_AATATAAT. For Haplo2308, animals with at least one copy of the ATAAT haplotype had a larger TNB and higher S/P ratio. Finally, the diplotype AAA_TTT for Haplo2273 had a smaller NBD than diplotypes TTT_AAA, TAA_TTT, and AAA_AAA. For S/P ratio, the diplotype TTT_TTT had a greater antibody response. Combining the results for NBD and S/P ratio, sows with the diplotype TTT_TTT are expected to have a better performance, with a smaller NBD and higher S/P ratio. These results provide further support that the genomic region on SSC7 encompassing the MHC region and its surrounding regions includes genomic variants (SNPs and/or haplotypes) that are at the same time associated with S/P ratio and reproductive performance. Moreover, specific diplotypes favorably associated with S/P ratio (i.e., higher S/P ratio) may also have a favorable impact on reproductive performance, such as increasing NBA and TNB while decreasing NBD.

## Conclusions

We have identified specific ROH and ROHet regions that are associated with S/P ratio, although the percentages of ROH and ROHet across the genome were not associated with this trait. The identified ROH regions may correspond to loci that are under selection in both parental lines, while the ROHet regions seem to be maintained by a heterozygote advantage at immune-related genes. The haplotype approach used in this study revealed novel genomic regions associated with S/P ratio on SSC4, 7, and 9. Some of the haplotype blocks located on SSC7 were also associated with reproductive performance (NBA, TNB, and NBD), supporting the use of S/P ratio as a selection tool to improve reproductive performance in vaccinated commercial sows. A potential QTL for S/P ratio in the MHC region appeared to be in stronger LD with the haplotype Haplo2293 (SSC7 ~ 25 Mb) than with the previously identified SNP located within this region. Furthermore, although the QTL appeared to be located in the MHC class II region, most likely, the causal gene(s) associated with this trait is(are) associated with antigen processing through the MHC class I, which is responsible for presenting endogenous antigens, such as viruses, to immune defense cells. *SLA-DOB* and *TAP2* are strong candidate genes within this region. The novel genomic regions that were identified to be associated with antibody response to PRRSV vaccination through the haplotype and heterozygosity and homozygosity analyses performed in our study provide additional resources for marker-assisted and genomic selection for improved response to PRRSV vaccination and reproductive performance in commercial pig populations. Additional analyses, such as whole-genome sequencing and proteomics could be helpful to identify the causal mutation associated with antibody response to PRRSV vaccination and, more specifically, with the correlation between antibody response to PRRSV vaccination and reproductive traits.

## Supplementary Information

**Additional file 1: Table S1.** ROH and ROHet identified in the study. Spreadsheets 1 and 2 contain results for ROH and ROHet, respectively. The columns represent: (1) ROH/ROHet numbered according to the number of SNPs within each ROH/ROHet; (2) identification of the individual; (3) chromosome where the ROH/ROHet is located; (4) SNP where the ROH/ROHet starts; (4) SNP where the ROH/ROHet ends; (5) position in kb where the ROH/ROHet starts; (6) position in kb where the ROH/ROHet ends; (7) size of the ROH/ROHet; and (8) number of SNPs within a ROH/ROHet for a given individual.

**Additional file 2: Table S2.** Associations of ROH/ROHet with S/P ratio. Spreadsheets 1 and 2 contain results for ROH and ROHet, respectively. The columns represent: (1) ROH/ROHet numbered according to the number of SNPs within each ROH/ROHet; (2) *P*-value for the association of ROH/ROHet with S/P ratio; (3) coefficient for the association of ROH/ROHet with S/P ratio (positive number indicate that the presence of ROH is associated with a greater S/P ratio); (4) standard error for the association of ROH/ROHet with S/P ratio.

**Additional file 3: Figure S1.** Relationship between percentage of ROH (a, c, and e) and ROHet (b and d) with antibody response [sample-to-positive (S/P) ratio] to porcine reproductive and respiratory syndrome virus vaccination. Based on the whole genome (a and b), *Sus scrofa* chromosome (SSC) 7 (c and d), and major histocompatibility complex (MHC) (e). The y-axis represents the adjusted S/P ratio, and the x-axis represents percentage of ROH or ROHet.

**Additional file 4: Table S3.** Haplotypes identified in the genome of commercial sows. The columns represent: (1) haplotype numbered by order of appearance along the genome; (2) number of SNPs composing the haplotype; (3) chromosome where the haplotype is located; (4) initial position of the haplotype within a chromosome; (5) end position of the haplotype within a chromosome; (6) length of the haplotype in kb; and (7) SNPs composing the haplotype.

**Additional file 5: Table S4.** Association of haplotypes with S/P ratio (approach 2). The columns represent: (1) haplotype numbered by order of appearance in the genome; (2) chromosome where the haplotype is located; (3) *P*-value for the association analyses; and (4) *q*-values for the association analyses (*P*-values corrected for multiple testing using false discovery rate method).

**Additional file 6: Table S5.** Effect of S/P ratio haplotypes on reproductive performance. The columns represent: (1) reproductive traits; (2) haplotype numbered according to their location along the genome; (3) haplotype; (4) expected mean of the reproductive traits for each diplotype; and (4) standard error of the mean.

## Data Availability

The data that support the findings of this study are not publicly available. Data may be available from authors upon request and authorization from the company that generated the data.
