# Supplementary figures and images for: Further host-genomic characterization of total antibody response to PRRSV vaccination and its relationship with reproductive performance in commercial sows: genome-wide haplotype and zygosity analyses
Source: Genet Sel Evol. 2021 Dec 7;53:91. doi: 10.1186/s12711-021-00676-5 (PMC8650375; doi:10.1186/s12711-021-00676-5)

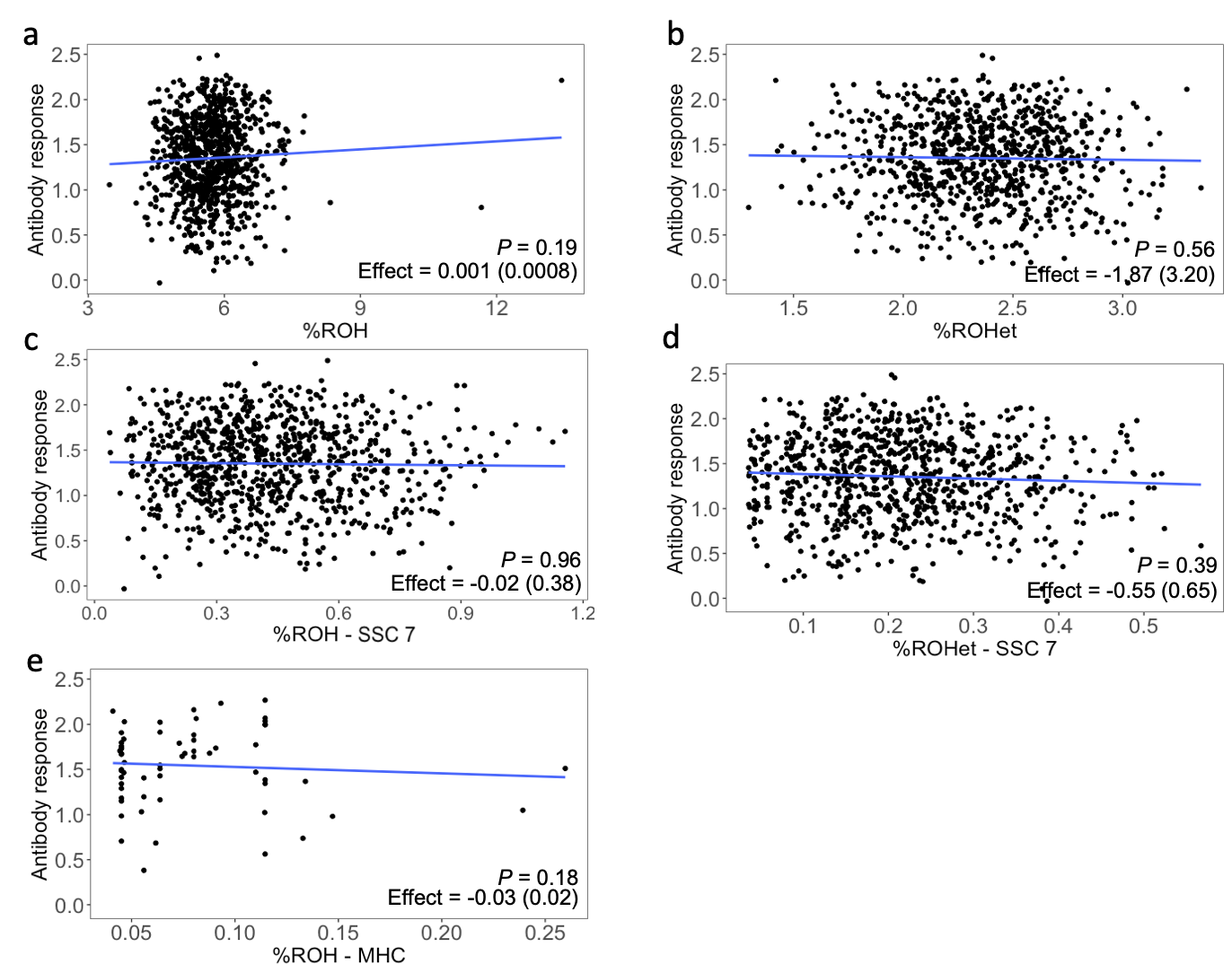

Supplement: Supplementary file 3 — Additional file 3: Figure S1. Relationship between percentage of ROH (a, c, and e) and ROHet (b and d) with antibody response [sample-to-positive (S/P) ratio] to porcine reproductive and respiratory syndrome virus vaccination. Based on the whole genome (a and b), Sus scrofa chromosome (SSC) 7 (c and d), and major histocompatibility complex (MHC) (e). The y-axis represents the adjusted S/P ratio, and the x-axis represents percentage of ROH or ROHet. [file 12711_2021_676_MOESM3_ESM.png]
